# Assessing the importance and feasibility of quality measures for chiropractic care: a national survey of U.S. chiropractors

**DOI:** 10.1186/s12998-026-00635-1

**Published:** 2026-03-28

**Authors:** Maranda Kleppe, Jacob McCarey, Sujatha Kedilaya, Robert Vining

**Affiliations:** Palmer Center for Chiropractic Research, Palmer College of Chiropractic, 1000 Brady St, Davenport, IA USA

**Keywords:** Quality, Chiropractic, Quality indicators, Quality improvement, Patient safety, Survey

## Abstract

**Background:**

Quality measures (QMs) are evidence-based metrics used to assess health care quality. A preliminary set for chiropractic care was recently developed and subsequently validated using modified Delphi consensus methodology. To prepare for eventual use, this study explored U.S. licensed chiropractors’ perceptions of the importance and feasibility of implementing quality measures in practice settings with distinct characteristics (e.g., single vs. multidisciplinary, portal-of-entry vs. referral access, varying population density regions, and health professional shortage areas).

**Methods:**

An electronic survey of U.S. licensed chiropractors was administered from March-May 2025. We mailed 22,330 postcard invitations and distributed electronic invitations through newsletters and/or social media announcements from chiropractic organizations and institutions. Each respondent rated 31 QMs of clinical processes and outcomes. QMs were rated on the importance of supporting quality care and the feasibility of implementation in respondent settings on a 5-point Likert scale (low to high importance/feasibility). We calculated mean ratings and rank-ordered measures, comparing subgroups differing by setting characteristics. We assessed rank-order correlation with Kendall’s Tau correlation coefficient, and rank-order agreement using Kendall’s W. To ensure results reflected national demographic proportions, responses were weighted on seven demographic variables, and analyses compared between weighted and unweighted data.

**Results:**

Eight hundred sixty-nine respondents completed the survey. Respondents were 69% male, with a mean age of 51.4 (SD = 14.3) years and a mean of 23.8 (SD = 14.1) years of experience. For importance ratings, rank-order correlation between subgroups was high (Kendall’s Tau = 0.75 or above) and agreement was unusually strong (Kendall’s W = 0.96). For feasibility ratings, correlation was moderate or higher (Kendall’s Tau = 0.55 or above) and agreement was strong (Kendall’s W = 0.89). ‘Documenting adverse events’ was the highest rated measure for importance with a mean of 4.79 (SD = 0.56) and ‘Performing an exam for a presenting problem’ was the highest rated measure for feasibility with a mean of 4.65 (SD = 0.77). Rank-order correlation and agreement from weighted and unweighted analyses were not appreciably different. Eight of the 10 highest-rated clinical process measures directly or indirectly support patient safety.

**Conclusions:**

Ratings for importance and feasibility differed little among subgroups defined by population density and health setting characteristics. The high importance ratings for safety-related clinical process measures are consistent with the concept of patient safety as a fundamental component of quality care.

**Supplementary Information:**

The online version contains supplementary material available at 10.1186/s12998-026-00635-1.

## Background

Quality measures (QMs) are evidence-based quantitative metrics used to assess health care quality across the continuum of care [1]. QMs are also used to support organizational accreditation, assess and improve care by defining quality targets, monitor performance and progress, and for public reporting [1, 2]. QMs encourage and facilitate the delivery of evidence-based care. However, until recently, there was no common set of QMs for chiropractic care published in peer-reviewed literature.

As a health profession, chiropractic has an obligation to continuously evaluate and improve quality in clinical care [3]. To facilitate quality assessment and improvement, Vining et al., developed a preliminary set of 70 QMs for chiropractic care derived from clinical guidelines, care standards, and best practices [4]. The next step in the development and validation process involved rating preliminary QMs by chiropractic clinicians in integrative settings and administrators overseeing chiropractic services with modified Delphi consensus methodology [5]. QMs achieving consensus were considered appropriate and relevant to patients, family members, clinicians, or health organizations. The next developmental step is to begin to prioritize QMs for eventual pilot implementation by assessing which are most important and theoretically feasible to implement.

Successful implementation of QMs requires in depth knowledge of a health care setting. Notable differences exist between settings that may influence quality assessment, such as whether chiropractic care is delivered in single discipline or multidisciplinary settings. Multidisciplinary health organizations typically have more resources and established processes needed for quality assessment compared to single discipline settings. In the U.S., multidisciplinary organizations also commonly obtain accreditation from external sources like the Joint Commission and the Accreditation Association for Ambulatory Health Care to objectively demonstrate evidence of high patient safety standards and a commitment to ongoing quality improvement. Chiropractic providers in multidisciplinary settings are therefore, more likely familiar with QM concepts [6, 7]. 

Another potentially influencing factor is how patients access chiropractic care. In most settings patients access care directly, whereas in others, access occurs primarily through referral from other providers. As a result, some clinical screening activities may be less essential when chiropractic care is accessed through referral by primary care providers who may routinely screen for some health factors. The regions in which patients access care may also influence which QMs are most important and feasible to implement. Patients in rural areas and areas with a shortage of health care providers have reduced access to services, potentially making screening activities more important in these regions. Currently, it is unknown if any of these setting characteristics have a major effect on perceived importance and feasibility of implementing QMs.

The long-term goal of this research is to facilitate evidence-based and standardized quality assessment for chiropractic care in a variety of settings where services are offered. This study focused on U.S. settings because differences in national health system characteristics can potentially affect the applicability of some QMs. U.S. health system characteristics that differ from most other countries include: [8]

- The absence of a central body governing health care delivery.
- Access largely based on insurance coverage.
- Individual health systems function under both market and non-market-like conditions (i.e., an imperfect market).
- Third party payers act as intermediaries.
- Multiple payers with different standards and requirements exist, potentially influencing quality-related clinical processes, priorities, and measurements.
- Legal risks influence practice behavior.

The purpose of this study was to conduct a nationally representative survey of U.S. licensed chiropractors to examine perceptions of the importance and feasibility of implementing QMs for chiropractic care. Specifically, we ranked QMs from respondent ratings on importance and feasibility and assessed if rank order differed substantially among: (A) single discipline vs. multidisciplinary settings; (B) settings where chiropractic care is primarily accessed through referral vs. settings where patients access care directly; (C) low population density areas vs. high population density areas; and (D) primary care health professional shortage areas (HPSAs) vs. areas without health professional shortages.

## Methods

### Overview

We conducted a cross-sectional electronic survey of licensed U.S. chiropractors. Ethics approval was reviewed and deemed exempt by the Palmer College of Chiropractic Institutional Review Board, Assurance # 2024-010. Respondents rated the relative importance and feasibility of implementing 41 QMs considering the specific setting in which they deliver care. The survey was administered in English and conducted from March 3, 2025, to May 30, 2025. This study is reported in accordance with the Consensus-Based Checklist for Reporting of Survey Studies (CROSS) guidelines [9]. 

### Survey platform

The survey was developed by the investigative team, then pretested by 4 licensed chiropractors who were not associated with the research. Feedback related to question clarity was incorporated into the final version. Respondents completed the anonymous survey through REDCap (Research Electronic Data Capture, Vanderbilt University, Nashville, TN). REDCap is a secure, web-based, data capture application. The survey was designed for data collection using a desktop or laptop computer, tablet, or mobile device. The URL and QR code located in recruitment messages (post cards, social media, newsletters) linked potential respondents to the consent document housed within REDCap. A reCAPTCHA feature preceded the consent document to ensure responses were completed by humans. Electronic consent was documented in REDCap by clicking ‘yes’ or ‘no’ under the “I consent to participate” statement. Denial of consent prevented respondents from completing the survey. No financial incentive was offered to complete the survey.

### Pre-recruitment activities

Eligible respondents self-identified as holding an active U.S. license to practice chiropractic and having current or prior experience in clinical practice. In the United States, licensees are required to complete a Doctor of Chiropractic degree through an accredited training program, pass a series of National Board Examinations, and fulfill additional training requirements mandated by the state or territory in which they are licensed (e.g., state laws, public health reporting requirements, ethics) [10]. To identify respondents most likely to meet inclusion criteria, we obtained publicly available lists of active licensees from state licensing boards including Washington DC. Lists were available for all states, except Oregon. Most board-provided lists included a mailing address unless licensees opted out. Seven states did not provide addresses: Arkansas, Delaware, Georgia, Hawaii, Illinois, New Hampshire, and Utah. For these states, and Oregon, we used state chiropractic association directories to manually identify licensees with mailing address information. Each member listed in a state association directory was then manually cross-checked with the respective state board licensee roster to verify active license status. After identifying 83,298 licensees from state board lists and 2219 from state association lists, we removed duplicates, those with disciplinary actions, non-U.S. addresses, and those with no address listed, resulting in 71,107 unique licensees with a postal address. Because QM ratings may differ for providers in different health settings and between regions with distinct population-based characteristics, we purposefully sought to identify respondents in pre-specified categories and population-based regions.

#### Access through referral

In 2 U.S. healthcare systems, patients access chiropractic care primarily through referral, Veteran’s Health Administration (VHA) and Military Treatment Facilities (MTF) [11]. VHA licensees were identified using the publicly available ‘VA Find a Healthcare Provider’ tool [12]. We then obtained mailing addresses for VHA licensees from lists generated from state boards and associations or from the parent facility location listed within the VHA Healthcare Provider profile. MTF provider names are not publicly listed so respondents were not further pre-identified.

#### Rural/small town areas

Zip codes associated with each licensee were used to pre-identify respondents located in rural/small town regions using Rural-Urban Commuting Area (RUCA) codes published by the U.S. Department of Agriculture. RUCA codes classify geographical areas based on population density, urbanization, and daily commuting patterns [13]. 

#### Health professional shortage areas (HPSAs)

Primary care Health Professional Shortage Areas (HPSAs) are regions with a population to provider ratio of at least 3500–1 or 3000–1 in areas with unusually high needs [14]. We identified licensees geographically located in primary care HPSA designated by the U.S. Heath Resources & Services Administration and Centers for Medicare and Medicaid Services using licensee zip codes [15, 16].

### Recruitment

We sent all licensees (*n* = 6542) located in rural/small town, HPSA, and/or patient access through referral settings two separate postcard invitations to complete the survey. Invitations were sent approximately 8 weeks apart. We also sent a single postcard invitation to those with a valid address (*n* = 15,788) generated from a 25% random sample of the remaining licensees stratified by geographic region (Northeast, Midwest, South, and West) as defined by the U.S. Census Bureau [17]. In total, postcard invitations were sent to 22,330 licensees. All postcard invitations included brief information about the study, an invitation to participate, and a custom shortened URL and QR code to access the survey.

To increase the number of potential respondents, we asked chiropractic organizations (*n* = 30), such as the American Chiropractic Association, chiropractic state associations (*n* = 59), and U.S. chiropractic educational institutions (*n* = 16) to distribute electronic survey invitations to members and alumni, via email, newsletter, and/or social media posts. Eleven organizations, 24 state associations, and 11 educational institutions agreed. Survey information was also posted twice in chiropractic-related Facebook groups (*n* = 10) reporting between 1200 and 12,000 members.

### Survey content

Two eligibility questions asking about current licensure status and experience in clinical practice were included at the beginning of the survey. Respondents answering ‘no’ to either question were thanked for their interest and excluded. Eligible respondents were then asked questions about their current or former primary practice setting including a description of the setting (i.e., single discipline or multidisciplinary), how patients access care (i.e., self-referral or referral only), and zip code of primary practice location. We defined single discipline settings as those where chiropractors were the highest-level providers and multidisciplinary settings as those including other primary care level providers such as MD, DO, DPT. We included nurse practitioners, physician assistants and occupational therapists in this definition because each profession engages in clinical activities overseen by or otherwise consistent with primary care providers with MD and DO degrees and/or engages in rehabilitation activities to enhance health for people with disability and non-disability related needs [18]. Survey questions are available in Supplemental file 1.

All respondents rated 31 QMs describing processes related to providing or receiving care and those measuring outcomes of care. Respondents who self-identified in a healthcare administrator role rated an additional 10 QMs describing general health organization characteristics that support quality care but do not directly or indirectly link to a clinical process or outcome measure (e.g., conducts regular compliance audits, costs of care are transparent). Respondents did not rate 23 previously developed QMs describing characteristics for which there is a matching process measure for 3 reasons. (1) To avoid potential confusion when rating QMs that could otherwise appear redundant; (2) Because corresponding process measures inherently presume organizational structure is present to support such measurement; and (3) To reduce respondent burden.

QMs were organized into 3 domains recommended by the Agency for Healthcare Research and Quality for public reporting and to aid in consumer understanding [19].

1. QMs supporting *care that gets results* (i.e., effective; efficient).
2. QMs supporting *care that protects patients from error and does not cause harm* (i.e., safe).
3. QMs supporting *care that is responsive to patient needs and preferences* (i.e., patient-centered, timely, and equitable).

QMs were presented in the same order for each respondent. All rating questions were included on one scrolling webpage (Supplemental file 1). Respondents rated all QMs for importance and feasibility. Importance was rated with the following question: ‘How important is this for supporting quality care in your setting.’ Feasibility was rated with the following questions: ‘How feasible is measuring this in your setting (e.g., % of patients, visits, or care plans)?’ for QMs describing clinical processes and outcomes, and ‘How feasible is developing or documenting this in your setting?’ for QMs describing characteristics of a health organization. All responses were rated on a scale of 1 (low importance) to 5 (high importance) or 1 (minimally feasible) to 5 (highly feasible).

### Statistical analysis

We considered respondents as duplicate when identical demographic data were reported. When this occurred, the first chronological survey completion was included for analysis while subsequent (i.e., duplicate) surveys were removed. After removing duplicates, all data were analyzed. For each QM, the mean rating was computed using all respondents with a non-missing response for that measure. Due to the high number of QMs, we used importance and feasibility mean ratings to rank order clinical process and outcome measures. Though the distributions of some QM rankings were skewed, rating methods using means is an appropriate ranking method in survey-based research [20, 21]. QMs describing health organization characteristics were ranked separately because only respondents self-reporting as an administrator rated them.

Kendall’s Tau-b correlation coefficient was used to assess the correlation of rank order of measures between pairs of respondent subgroups differentiated by health organization characteristics and population-based regions [22]. Correlation coefficients were interpreted as negligible (0.00–0.30), low (0.30–0.50), moderate (0.50–0.70), high (0.70–0.90), and very high (0.90−1.00) [23]. To assess agreement among ranked lists between 3 or more groups, we used Kendall’s W and interpreted values as no agreement (0.00–0.09), very weak (0.10–0.29), weak (0.30–0.49), moderate (0.50–0.69), strong (0.70–0.89), and unusually strong (0.90−1.00) [22]. Specifically, we compared ranked lists of measures between respondents in single discipline, multidisciplinary, and referral-only settings.

For each estimate, we calculated bias-corrected and accelerated (BCa) bootstrap 95% confidence intervals. BCa bootstrapping was chosen because of its ability to accommodate the non-parametric and bounded nature of the Kendall’s Tau-b and Kendall’s W statistics. A sensitivity analysis compared results of unweighted data to results after using iterative proportional fitting (raking) to adjust survey weights so that the sample’s marginal proportions would match the estimated demographic proportions of chiropractors nationally [24]. Data was weighted on age group, gender, race, practice setting, population density, HPSA status, and geographic region according to estimates from prior national chiropractic surveys [10, 25]. All analyses were conducted using R (R version 4.3.1, Vienna, Austria) and SAS (version 9.4, SAS Institute Inc., Cary, NC).

## Results

Twelve surveys containing identical demographic data were identified as duplicates and removed from the final dataset, leaving 869 for analysis. Most respondents learned of the survey from a newsletter or email (62%, *n* = 536), or postcard (23%, *n* = 203). The mean (SD) respondent age was 51 (14.3) years, ranging from 24 to 86, with 69% male (*n* = 597). Mean licensure duration was 24 (14.1) years. Most respondents (43%, *n* = 369) reported engaging in clinical activities between 30 and 39 h per week. Respondents graduated from 17 different training programs located in the continental U.S., Puerto Rico, and Canada. 8% (*n* = 71) of respondents held administrative responsibilities overseeing care. Additional demographic data are presented in Table 1.

**Table 1:** Respondent demographics

Age (y) (mean (SD)), range	51.4 (14.3), 24–86	
Gender		
Male	597 (68.7)	
Female	258 (29.7)	
Other	3 (0.3)	
Not specified	11 (1.3)	
Race		
American Indian or Alaskan Native	3 (0.4)	
Asian	5 (0.6)	
Black or African American	14 (1.6)	
Native Hawaiian or Other Pacific Islander	1 (0.1)	
White	798 (91.8)	
Multiracial	13 (1.5)	
Not specified	35 (4.0)	
Ethnicity		
Hispanic or Latino	28 (3.2)	
Not specified	84 (9.7)	
Additional professional/academic degrees		
Masters	80 (9.2)	
PhD	10 (1.2)	
Other (e.g., PT, MD, ND, PharmD)	9 (1.0)	
Chiropractic training institution		
Cleveland University Kansas City (including Cleveland Chiropractic College of L.A.)	41 (4.7)	
Life University (including Life Chiropractic College West)	165 (19.0)	
Logan University	82 (9.4)	
National University of Health Sciences (including Illinois and Florida campuses)	55 (6.3)	
Northeast College of Health Sciences	77 (8.9)	
Northwestern Health Sciences University	61 (7.0)	
Palmer College of Chiropractic (including Main, Florida, and West campuses)	274 (31.5)	
Parker University	23 (2.6)	
Sherman College of Chiropractic	16 (1.8)	
Southern California University of Health Sciences	22 (2.5)	
Texas Chiropractic College	14 (1.6)	
University of Bridgeport	7 (0.8)	
University of Western States	24 (2.8)	
Other	4 (0.5)	
Not specified	4 (0.5)	
Years actively licensed (mean (SD))	23.8 (14.1)	
Current professional role*		
Clinician	834 (96.0)	
Educator	126 (14.5)	
Administrator	71 (8.2)	
Researcher	39 (4.5)	
Not currently practicing	4 (0.5)	
How respondents learned about the survey*		
Newsletter/email	536 (61.7)	
Social media	106 (12.2)	
Postcard	203 (23.4)	
Friend/colleague	35 (4.0)	

n (%) unless otherwise noted; *Total may exceed 100% because respondents could select more than one option

Most respondents (84%, *n* = 728) reported working in single discipline settings, while 16% (*n* = 141) reported working in multidisciplinary settings. 71% of those in multidisciplinary settings reported working with medical doctors, 67% with physical therapists, 60% with nurse practitioners, 48% with doctors of osteopathy, 47% with physician assistants, and 28% with occupational therapists. The majority of respondents (94%, *n* = 818) were located in settings where patients could access chiropractic care without a referral, while 6% (*n* = 49) reported access through referral-only. Sixty-six percent of respondents in multidisciplinary settings reported being familiar with QMs compared to 39% in single discipline settings. Characteristics of respondent settings are further described in Table 2.

**Table 2:** Characteristics of respondent settings

	*n* (%)
Practice setting	
Single discipline	728 (83.8)
Multidisciplinary	141 (16.2)
Access to care	
Self-referral	818 (94.1)
Referral required	49 (5.6)
Not specified	2 (0.2)
Population density	
Metropolitan/micropolitan	723 (83.2)
Rural/small town	121 (13.9)
Not specified	25 (2.9)
HPSA*	
No	770 (88.6)
Yes	74 (8.5)
Not specified	25 (2.9)
U.S. geographic region	
Midwest	300 (34.5)
South	245 (28.2)
Northeast	161 (18.5)
West	138 (15.9)
Not specified	25 (2.9)

*Health professional shortage area

The sensitivity analysis using QM ratings weighted on respondent demographic variables demonstrated a similar rank order of measures with unweighted data. Measures of correlation between subgroups (e.g., rural or HSPA location, single discipline and multidisciplinary settings) were also similar for both the weighted and unweighted data, and interpretations of correlation coefficients remained consistent. We report unweighted results because they are based on a more straightforward statistical analysis and are, therefore, more transparent.

Overall importance and feasibility rankings for all QMs are displayed in Tables 3 and 4. With a maximum score of 5, indicating high importance for supporting quality care, the highest rated process measure was ‘Documenting a condition-specific history’ with a mean of 4.79 (SD = 0.54). The highest rated process QM for feasibility was ‘Performing an exam for a presenting problem’ with a mean of 4.65 (SD = 0.77). The highest rated outcome measure for both importance and feasibility was ‘Documenting adverse events,’ with mean ratings of 4.79 (SD = 0.56) and 4.53 (SD = 0.90) respectively. The highest rated characteristic of a health organization for both importance and feasibility was ‘Securing patient records according to regulatory requirements’ with mean ratings of 4.74 (SD = 0.76) and 4.61 (SD = 0.86), respectively. Of the 41 QMs rated, all received a mean rating for importance and feasibility of above 3.25. Eight of the 10 highest-rated (for importance) clinical process measures promote patient safety, 4 directly and 4 indirectly. The highest rated outcome measure and health organization characteristic also directly support patient safety. Supplemental file 2 lists all 64 current QMs.

**Table 3:** Importance rankings for quality measures of clinical processes, care outcomes, and health organization characteristics^†^

Measures of clinical processes	Mean rating (SD)
1. Documenting a condition-specific history	4.79 (0.54)
2. Performing an exam for a presenting problem	4.79 (0.60)
3. Screening for signs and symptoms of serious pathology (i.e. red flags)	4.72 (0.66)
4. Completing an informed consent process before delivering care	4.69 (0.78)
5. Care plans are based on a clinical evaluation	4.69 (0.71)
6. Referring patients with new/recent osteoporotic fracture to a primary care or other relevant provider	4.65 (0.84)
7. Regularly assessing response to care	4.62 (0.72)
8. Documenting a past health history	4.61 (0.71)
9. Care plans include: (1) Active therapies such as supervised or unsupervised exercise; (2) Manual therapies such as joint manipulation and myofascial therapies; (3) Education about one’s condition including pain physiology when appropriate; (4) Self-management advice and/or activities; and (5) Therapeutic goals. Note: All components are not required at each visit.	4.56 (0.90)
10. Screening for the possibility of pregnancy prior to obtaining radiographs	4.42 (1.20)
11. Each visit is conducted as part of a current care plan	4.42 (0.90)
12. Offering older adults advice on balance, strength, and endurance exercises to prevent falls	4.35 (0.98)
13. Referring patients at risk for self-directed violence to an appropriate provider	4.32 (1.08)
14. Number of days to obtain an appointment for chiropractic care	4.30 (1.12)
15. Assessing the need for additional visits at each visit	4.19 (1.11)
16. Documenting patient involvement in care planning and decision-making	4.18 (1.12)
17. Assessing patients with valid functional and/or symptom outcome measures at baseline	4.16 (1.05)
18. Screening for physical activity level	3.95 (1.05)
19. Documenting a review of systems (e.g., cardiovascular, pulmonary, etc.)	3.95 (1.08)
20. Screening older adults for abilities to independently carry out activities of daily living	3.92 (1.17)
21. Documenting a current medication list	3.85 (1.21)
22. Screening patients over age 40 for major risk factors for osteoporosis	3.72 (1.21)
23. Recording vital signs	3.61 (1.33)
24. Screening for opioid use	3.54 (1.35)
25. Screening for psychological and social risk factors	3.52 (1.17)
26. Screening for tobacco use	3.25 (1.43)

Measures of care outcomes	Mean rating (SD)
1. Documenting adverse events	4.79 (0.56)
2. Patients report satisfaction with care	4.71 (0.66)
3. Assessing patients with valid functional and/or symptom outcome measures during a re-evaluation	4.28 (0.98)
4. Patients report involvement in care planning and decision-making	4.28 (1.02)
5. Return to work time for patients with a work-related injury	4.20 (1.12)

Measures of health organization characteristics	Mean rating (SD)
1. Securing patient records according to regulatory requirements	4.74 (0.76)
2. Costs of chiropractic care are transparent	4.63 (0.82)
3. General employment training procedures	4.41 (0.97)
4. A current database of professional credentials for all providers	4.36 (1.08)
5. A reporting/supervisory structure	4.30 (1.00)
6. A future planning strategy	4.29 (0.92)
7. Training procedures for hand hygiene, protective equipment, and environmental cleaning	4.23 (1.22)
8. Conducting regular audits are part of a quality control program	4.17 (1.15)
9. Conducting regular audits to ensure regulatory compliance	4.13 (1.17)
10. Infection control and prevention protocols	4.03 (1.30)

^†^Rounding resulted in mean ratings to appear equivalent; additional decimal places revealed differences in rank order

**Table 4:** Feasibility rankings for quality measures of clinical processes, care outcomes, and health organization characteristics^†^

Measures of clinical processes	Mean rating (SD)
1. Performing an exam for a presenting problem	4.65 (0.77)
2. Completing an informed consent process before delivering care	4.64 (0.81)
3. Documenting a condition-specific history	4.55 (0.88)
4. Care plans are based on a clinical evaluation	4.46 (0.93)
5. Screening for signs and symptoms of serious pathology (i.e. red flags)	4.45 (0.89)
6. Documenting a past health history	4.39 (0.95)
7. Regularly assessing response to care	4.38 (0.96)
8. Referring patients with new/recent osteoporotic fracture to a primary care or other relevant provider	4.36 (1.07)
9. Care plans include: (1) Active therapies such as supervised or unsupervised exercise; (2) Manual therapies such as joint manipulation and myofascial therapies; (3) Education about one’s condition including pain physiology when appropriate; (4) Self-management advice and/or activities; and (5) Therapeutic goals. Note: All components are not required at each visit.	4.29 (1.07)
10. Each visit is conducted as part of a current care plan	4.27 (1.03)
11. Screening for the possibility of pregnancy prior to obtaining radiographs	4.13 (1.36)
12. Assessing the need for additional visits at each visit	4.12 (1.13)
13. Number of days to obtain an appointment for chiropractic care	4.08 (1.22)
14. Documenting patient involvement in care planning and decision-making	4.07 (1.15)
15. Assessing patients with valid functional and/or symptom outcome measures at baseline	4.04 (1.10)
16. Offering older adults advice on balance, strength, and endurance exercises to prevent falls	4.01 (1.17)
17. Documenting a current medication list	3.94 (1.19)
18. Documenting a review of systems (e.g., cardiovascular, pulmonary, etc.)	3.89 (1.19)
19. Recording vital signs	3.86 (1.27)
20. Screening for physical activity level	3.80 (1.16)
21. Referring patients at risk for self-directed violence to an appropriate provider	3.80 (1.29)
22. Screening for tobacco use	3.76 (1.38)
23. Screening older adults for abilities to independently carry out activities of daily living	3.59 (1.23)
24. Screening for opioid use	3.51 (1.41)
25. Screening patients over age 40 for major risk factors for osteoporosis	3.33 (1.32)
26. Screening for psychological and social risk factors	3.31 (1.23)

Measures of care outcomes	Mean rating (SD)
1. Documenting adverse events	4.53 (0.90)
2. Patients report satisfaction with care	4.39 (0.99)
3. Assessing patients with valid functional and/or symptom outcome measures during a re-evaluation	4.07 (1.09)
4. Patients report involvement in care planning and decision-making	4.02 (1.17)
5. Return to work time for patients with a work-related injury	3.92 (1.18)

Measures of health organization characteristics	Mean rating (SD)
1. Securing patient records according to regulatory requirements	4.61 (0.86)
2. Costs of chiropractic care are transparent	4.36 (1.16)
3. Training procedures for hand hygiene, protective equipment, and environmental cleaning	4.28 (1.07)
4. A current database of professional credentials for all providers	4.25 (1.20)
5. General employment training procedures	4.16 (1.09)
6. A reporting/supervisory structure	4.12 (1.15)
7. Infection control and prevention protocols	3.90 (1.32)
8. Conducting regular audits to ensure regulatory compliance	3.90 (1.27)
9. Conducting regular audits are part of a quality control program	3.88^‡^ (1.27)
9. A future planning strategy	3.88^‡^ (1.21)

^†^Rounding resulted in mean ratings to appear equivalent; additional decimal places revealed differences in rank order

^‡^Ratings were identical

### Subgroup analysis

Table 5 reports the correlation of QM ratings between respondent subgroups. Pairwise comparisons of importance rankings for all subgroups resulted in a Kendall’s Tau value of 0.75 or above (high correlation). Feasibility comparisons were either highly or moderately correlated (0.55 or above) between subgroups. Agreement between single discipline, multidisciplinary, and referral only settings for 31 process and outcome measure ratings using Kendall’s W (95% CI) was unusually strong at 0.96 (0.73–1.00) for importance, and strong at 0.89 (0.66–1.00) for feasibility. For all subgroups, the mean importance ratings of all rated measures combined were higher (4.11–4.49) than mean feasibility ratings (3.97–4.31). Supplemental file 3 contains the mean ratings for QMs by subgroup for importance and feasibility and Kendall’s W calculations.

**Table 5:** Subgroup comparisons of importance and feasibility rankings using Kendall’s Tau correlation coefficients

Subgroups compared	Kendall’s Tau* (95% CI)
Importance	
Single discipline vs. Multidisciplinary	0.79 (0.68–0.87)
Single discipline vs. Referral only	0.75 (0.64–0.84)
Multidisciplinary vs. Referral only	0.83 (0.73–0.91)
Self-Referral vs. Referral only	0.76 (0.65–0.85)
Small town/Rural vs. Micro/metropolitan	0.83 (0.69–0.91)
HPSA^†^ vs. Non-HPSA	0.81 (0.67–0.89)
Feasibility	
Single discipline vs. Multidisciplinary	0.71 (0.47–0.83)
Single discipline vs. Referral only	0.55 (0.34–0.70)
Multidisciplinary vs. Referral only	0.77 (0.63–0.86)
Self-Referral vs. Referral only	0.55 (0.35–0.70)
Small town/Rural vs. Micro/metropolitan	0.83 (0.69–0.91)
HPSA vs. Non-HPSA	0.80 (0.64–0.90)

*Kendall’s Tau categories: negligible (0.00–0.30), low (0.30–0.50), moderate (0.50–0.70), high (0.70–0.90), and very high (0.90–.00); ^†^Health professional shortage area

## Discussion

This is the first study assessing U.S. licensed chiropractor’s perspectives on the importance and feasibility of implementing a systematically developed set of evidence-based QMs for chiropractic care. Despite the diverse population-based and organizational settings of respondents, there was moderate to high agreement for both importance and feasibility rankings between all subgroups. Five clinical process measures were rated highest for both importance and feasibility. Screening measures for opioid use, tobacco use, and psychological and social risk factors were consistently rated lowest. The lowest mean rating for any QM was 3.25, higher than the midpoint on a scale where 5 was the highest possible rating.

### Pilot implementation considerations

For each respondent subgroup, mean importance ratings (4.11–4.49) were higher than mean feasibility ratings (3.96–4.31). Importance ratings likely reflected conceptual agreement with QMs whereas slightly lower feasibility ratings may have reflected an awareness that adding measurement processes can be challenging. 17% of respondents reported not being familiar with QMs, with 39% reporting some familiarity. The relative lack of familiarity, together with the absence of widespread use of QMs in chiropractic practice, may have inflated feasibility ratings. Respondents may have also presumed data collection from individual file reviews rather than automated processes, further influencing feasibility ratings. Ideally, QMs are obtained from administrative data to avoid auditor bias, strained personnel resources, and challenges with inadequate sample sizes associated with file-review processes [26, 27]. 

Successful QM implementation requires infrastructure, policies, and processes to support data collection, analysis, and response cycles [28]. Successful implementation also requires an organizationally tailored approach and pilot testing. Based on data from this study, pilot QMs to consider are the 5 clinical processes, 3 outcomes, and 2 health organization characteristics rated highest for both importance and feasibility.

QMs of clinical processes:

- Documenting a condition-specific history.
- Performing an exam for a presenting problem.
- Screening for signs and symptoms of serious pathology.
- Completing an informed consent process before delivering care.
- Basing care plans on a clinical evaluation.

QMs of outcomes:

- Documenting adverse events.
- Patient satisfaction with care.
- Assessing patients with a valid functional and/or symptom outcome measure at re-evaluation.

QMs of health organization characteristics:

- Securing patient records according to regulatory requirements.
- Costs of chiropractic care are transparent.

The Institute for Healthcare Improvement offers systematic guidance for those seeking to implement QMs [29, 30]. Resources include toolkits, videos, and guidance on topics such as forming teams to adequately support QM use, conducting quality improvement cycles, and implementing data informed changes. Though good clinical outcomes are the ultimate goals of care, evidence-based clinical processes and organizational characteristics linked to improved outcomes are critical to measure. Outcome data cannot be meaningfully interpreted without knowing key organizational features and if clinical processes supporting safety and other outcomes are systematically performed. Without this knowledge, determining how to improve outcomes is difficult, at best [1]. Ensuring health organizations have the appropriate infrastructure to support quality measurement should be a major focus for those seeking to measure, assess, and improve the quality of care [28]. Without the infrastructure to support efficient, accurate, and sustainable data collection, successful QM implementation is unlikely. For example, implementing too many QMs at one time or implementing without sufficient infrastructure carries the potential to impede clinical workflows, lengthen visits, burden patients, providers, and staff, distract clinical focus, and reduce morale. Negative consequences from ineffective implementation can reduce the quality of care offered [31, 32]. To aid those seeking pilot implementation, Supplemental file 2 includes the current set of 64 QMs with rationale supporting each measure, example metrics, and a crosswalk linking clinical processes and outcomes with supporting structural measures (i.e., health organization characteristics).

### Direct influences on patient safety

Documenting adverse events was the highest rated measure for importance, suggesting patient safety is a primary consideration for practitioners. However, widespread adoption of adverse event documentation presents several challenges beginning with the need for standard definitions, data collection, and reporting practices. Simply adopting definitions, practices, and classification systems used in clinical trials may not be feasible. For example, in clinical trials, symptoms are often highly monitored over the course of a trial and adverse events are recorded and classified even when unrelated to study participation [33]. Further, distinguishing causal relationships can be challenging without control groups. In a randomized trial studying spinal manipulation for low back pain, 106 events were reported in the spinal manipulation group [34]. The 92 events occurring in the sham group suggested many events recorded for the spinal manipulation group were related to natural symptom variation rather than the intervention [34, 35]. Other challenges with documenting adverse events include addressing concerns about time constraints, patient perception, and potential legal consequences [36]. 

Potential evolving solutions to some of these challenges include 2 distinct methods to identify and report adverse events. The Chiropractic Patient Incident Reporting and Learning System (CPiRLS), an anonymous, online platform for reporting incidents that result in harm or potential harm, is available in the United Kingdom, Europe, and Australia [37]. CPiRLS is a passive system, relying on voluntary reporting by providers. In contrast, active surveillance methods involve proactive processes to identify potential adverse events. Early efforts piloting active surveillance methods have been reported as feasible in chiropractic teaching clinics and community-based settings [38, 39]. Active surveillance methods offer the potential for more accurate estimates of adverse event rates, though they are more resource-intensive [40]. 

In addition to documenting adverse events, other highly rated QMs directly relate to maintaining or improving safety. For example, QMs reflecting decision-making that reduces risk to patients include red flag screening, vital sign recording, performing exams for a presenting problem, regularly assessing response to care, and screening for pregnancy prior to obtaining radiographs. Health organization characteristics (i.e., structural QMs) also focus on safety by minimizing harm, such as establishing infection control protocols, and securing patient records.

### Indirect influences on patient safety

**Fig. 1:**
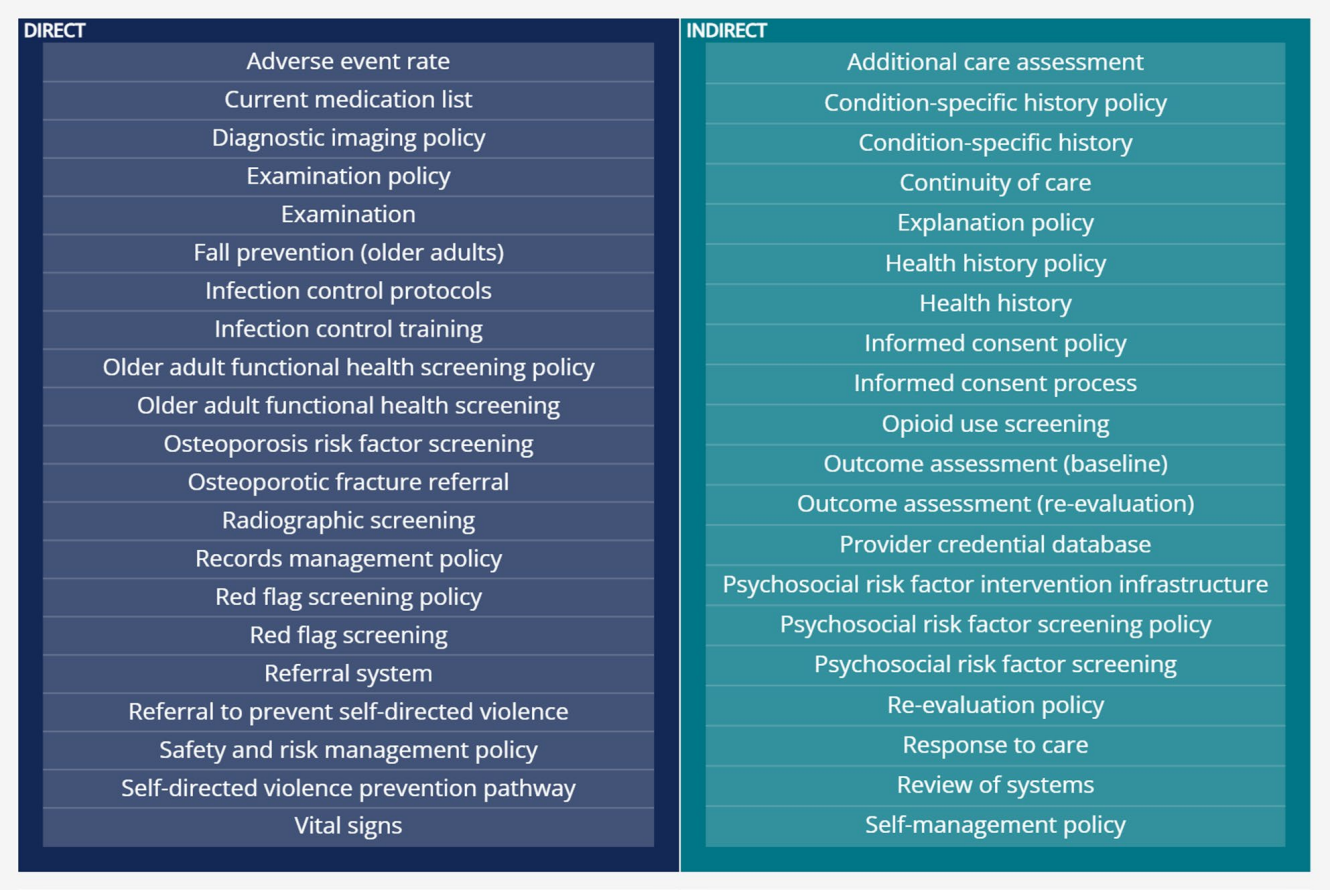
Quality measures directly and indirectly supporting patient safety. Direct: Measures that primarily focus on minimizing harm; Indirect: Measures that can contribute to safer care by identifying underlying risks or conditions necessitating referral, promoting patient comprehension and autonomy, and ensuring care aligns with individual patient needs

Although documenting adverse events and measures related to clinical decision-making may be the most intuitively related to patient safety, additional QMs indirectly support care that prevents patient harm. For example, QMs related to clinical outcomes are primarily focused on measuring the effectiveness of care. However, assessing treatment response also provides objective data about when care is no longer needed, indirectly reducing risks associated with receiving unnecessary treatment. Likewise, continuity of care between a patient and specific provider is intended to reduce waste, costs, and improve patient satisfaction [41]. Continuity of care is also associated with reduced all-cause mortality, hospitalizations, and emergency department utilization in primary care and specialist settings [42–45]. Fig. 1 displays 41 of 64 (64%) current QMs which either directly promote or indirectly support the concept of safety.

### Safety as a fundamental component of quality

High ratings of QMs associated with safety suggests respondents were conceptually aligned with the World Federation of Chiropractic-Global Patient Safety Initiative, which focuses on supporting a patient safety culture across the profession [46]. Rather than solely focusing on adverse event reporting, patient safety culture refers to shared values, attitudes, and behaviors related to minimizing patient harm [47, 48]. The initiative is informed by the World Health Organization Global Patient Safety Action Plan 2021–2030 [46]. Current QMs related to patient safety can provide objective data supporting at least 4 of the 7 strategic objectives included in the Action Plan’s Framework for Action [49]. 

Safety is a fundamental requirement of quality care. QMs offer an opportunity to systematically measure features of health organizations, clinical processes, and clinical outcomes, providing objective data to identify strengths and address challenges [2]. QMs can influence provider behaviors by facilitating and ensuring clinical processes are more systematically performed while simultaneously communicating safety is a priority for health organizations.

### Future directions

Chiropractic QM development has thus far included researchers and 2 iterations of review by U.S. – based providers, including administrators overseeing chiropractic care for a variety of health organizations. Perspectives from providers outside the U.S and from other relevant groups is needed. Most critically, patient input has not yet been formally included. As the recipients of care, patient input is essential for a comprehensive QM development process. Such input is also needed before a minimum standard set of QMs can be formally recommended. The Agency for Healthcare Research and Quality developed 13 person-centered QMs related to timeliness, provider communication, care coordination, respectful care, and provider ratings [50]. Future research will assess if the measures, which were validated in a variety of medical settings, are transferrable to chiropractic settings.

### Limitations

Because survey invitations included a publicly accessible web-link, duplicate responses from some respondents were possible. We used demographic information to identify and remove likely duplicate responses to minimize this potential. It was not possible to calculate an overall response rate due to overlapping recruitment strategies and an unknown reach through invitations shared through social media and announcements from educational institutions and professional organizations. Though postcard invitations achieved a calculated 1% response rate, potential cross-exposure to electronic announcements likely renders this estimate inaccurate. We achieved fewer responses compared to other recent national surveys. However, all surveys are potentially limited by response rates and the sample size of 869 was robust enough to ensure the number of respondents in each group supported stable mean ratings. We also weighted responses based on seven demographic variables obtained from larger and recent nationally representative surveys. Results from weighted and unweighted analyses were not appreciably different, suggesting a low risk of responder bias. Nevertheless, results may not reflect perspectives of the entire U.S chiropractic profession.

All respondents held licenses in the U.S. Thus, perspectives from providers outside the U.S. were not included in this study, potentially limiting results. Several of the highest rated items reflect measures to ensure patient safety. We anticipate provider perspectives from outside the U.S. would be similar because safety is a fundamentally important consideration for health professionals regardless of national or cultural perspective. Each respondent rated each measure for importance and feasibility using the same item sequence. Question sequencing was not randomized to ensure QM topic categories were organized and to prevent confusion. Nevertheless, sequencing could have influenced ratings. Providers are typically not involved in developing, managing, or assessing health organization infrastructure unless they serve an administrative role. Therefore, we asked only administrator respondents to rate QMs measuring health organization characteristics. Further, we did not include all QMs of health organization characteristics in the survey. Instead, only administrator respondents rated 10 QMs of characteristics which did not support any specific clinical process or outcome. We did not include QMs describing policies or infrastructure supporting clinical process and outcome QMs to avoid perceived redundancy and because QMs of clinical processes and outcomes presume infrastructure is in place to support them. The result of these combined factors was a clearer and far more efficient survey, though fewer respondents rated the 10 general QMs of health organization characteristics.

## Conclusion

The importance and feasibility of QMs rated in this study differed little despite differences in respondent settings defined by population density and other health setting characteristics. There was a high correlation for importance rankings for all subgroups while agreement between single discipline, multidisciplinary, and referral only settings was unusually strong for importance, and strong for feasibility. Rank-order correlation and agreement were also similar across weighted and unweighted analyses.

Five QMs were rated highest for both importance and feasibility. The highest rated measure for importance was documenting adverse events. Together with other highly rated measures that directly and indirectly contribute to preventing harm, QM ratings of survey respondents are consistent with the concept that safety is an essential component of quality chiropractic care.

## Supplementary Information

Below is the link to the electronic supplementary material.

Supplementary Material 1: Survey.

Supplementary Material 2: QM descriptions, metrics, rationale, and companion measures

Supplementary Material 3: Supplemental tables

## Data Availability

The datasets generated and/or analyzed during the current study are the property of Palmer College of Chiropractic and not publicly available. However, datasets are available from the corresponding author on reasonable request.

## Abbreviations

QM: Quality measure
HPSA: Health professional shortage area
CROSS: Consensus-based checklist for reporting of survey studies
REDCap: Research electronic data capture
VHA: Veterans health administration
MTF: Military treatment facility
RUCA: Rural-urban commuting area
CPiRLS: Chiropractic patient incident reporting and learning system
