# Supplementary material for: Assessing the importance and feasibility of quality measures for chiropractic care: a national survey of U.S. chiropractors
Source: Chiropr Man Therap. 2026 Mar 28;34:18. doi: 10.1186/s12998-026-00635-1 (PMC13151159; doi:10.1186/s12998-026-00635-1)
Supplement: Supplementary file 1 — Supplementary Material 1: Survey. [file 12998_2026_635_MOESM1_ESM.pdf]

Demographics

Do you hold an active U.S. license to practice chiropractic?

- (●) Yes
- ( ) No

reset

Do you have experience in clinical practice?

- (●) Yes
- ( ) No

reset

Please answer the following questions about your current (or former) primary practice setting:

What best describes the setting?

- (●) Multidiscipline *(Settings with other primary care level (or higher) providers such as MD, DO, DPT)*
- ( ) Single discipline *(Settings with only chiropractors as the highest-level providers)*

reset

What other provider types work in your setting?
(Mark all that apply):

- [ ] DO
- [ ] MD
- [ ] PT/DPT
- [ ] OT/OTD
- [ ] PA
- [ ] NP/CNP/DNP
- [ ] Other

How do patients primarily access chiropractic care?

- ( ) Patients can access care directly (self-referral)
- ( ) Referral from another medical professional is required

reset

Zip code of your primary practice location

[ ]

5-digit ZIP code (U.S.)

Which best characterizes your current professional role? *(Mark all that apply)*:

- [ ] Clinician: Actively provide patient care
- [ ] Educator
- [ ] Administrator: Oversee chiropractic services in educational or multidisciplinary settings (e.g., MD, DO, DPT, DC)
- [ ] Researcher
- [ ] Other:

How many hours per week are you typically engaged in clinical activities?

- ( ) Fewer than 9
- ( ) 10-19
- ( ) 20-29
- ( ) 30-39
- ( ) 40-49
- ( ) 50-59
- ( ) 60 or more

reset

Do you use an electronic health record (EHR) system?

- ( ) Yes
- ( ) No

reset

Are you familiar with quality measures used in healthcare settings? (e.g., emergency room wait times)

- ( ) Yes
- ( ) No
- ( ) Somewhat

reset

Age:

[in yrs]

Race *(Mark all that apply)*:

- [ ] American Indian or Alaska Native
- [ ] Asian
- [ ] Black or African American
- [ ] Native Hawaiian or Other Pacific Islander
- [ ] White
- [ ] Unknown

Ethnicity:

- [ ] Hispanic or Latino
- [ ] Not Hispanic or Latino
- [ ] Unknown

Gender *(Mark all that apply)*:

- [ ] Female
- [ ] Male
- [ ] Transgender
- [ ] Non-binary
- [ ] Other

Professional and/or academic degree *(Mark all that apply)*:

- [ ] DC
- [ ] PhD
- [ ] Masters
- [ ] PT
- [ ] DPT
- [ ] MD
- [ ] DO
- [ ] Other

Chiropractic training institution:

[▾]

Years actively licensed:

[in yrs]

How did you hear about this survey? *(Mark all that apply)*

- [ ] Newsletter/email
- [ ] Facebook
- [ ] Postcard
- [ ] Friend/colleague
- [ ] State Association
- [ ] Other

Quality Measures

A A A

Instructions

- Rate the following quality measures according to:
  - How important each is for demonstrating or supporting quality care, and
  - How feasible it would be to measure in your practice setting. (e.g., calculating the percentage of patients, visits, or care plans aligning with each measure)
- Responses save only after clicking the submit button at the bottom of the page.
- Note: The measures below are still developing. More research is needed to fully validate a comprehensive set.
- This survey will remain open until **5/30/2025**.
- Buttons at top right change text size.

This section contains 15 quality measures supporting care that gets results.

	How **IMPORTANT** is this for supporting quality care in your setting? 1=Low, 5=High	How **FEASIBLE** is **measuring** this in your setting? (*e.g., % of patients, visits, care plans*) 1=Minimally, 5=Highly
**Clinical Evaluation**		
Documenting a past health history	1 2 3 4 5	1 2 3 4 5
Documenting a condition-specific history	1 2 3 4 5	1 2 3 4 5
Documenting a review of systems (e.g., cardiovascular, pulmonary, etc.)	1 2 3 4 5	1 2 3 4 5
Assessing patients with valid functional and/or symptom outcome measures at baseline	1 2 3 4 5	1 2 3 4 5
Regularly assessing response to care	1 2 3 4 5	1 2 3 4 5
Assessing the need for additional visits at each visit	1 2 3 4 5	1 2 3 4 5
**Clinical Screening**		
Screening for physical activity level	1 2 3 4 5	1 2 3 4 5
Screening for psychological and social risk factors	1 2 3 4 5	1 2 3 4 5
Screening for tobacco use	1 2 3 4 5	1 2 3 4 5
Screening for opioid use	1 2 3 4 5	1 2 3 4 5
**Care Planning**		
Care plans are based on a clinical evaluation	1 2 3 4 5	1 2 3 4 5
Each visit is conducted as part of a current care plan	1 2 3 4 5	1 2 3 4 5
Care plans include: 1) Active therapies such supervised or unsupervised exercise; 2) Manual therapies such as joint manipulation, and myofascial therapies; 3) Education about one's condition including pain physiology when appropriate; 4) Self-management advice and/or activities; and 5) Therapeutic goals. *All components are not required at each visit.	1 2 3 4 5	1 2 3 4 5
**Clinical Outcomes**		
Assessing patients with valid functional and/or symptom outcome measures during a re-evaluation	1 2 3 4 5	1 2 3 4 5
Return to work time for patients with a work-related injury	1 2 3 4 5	1 2 3 4 5

This section contains 11 quality measures supporting care that protects patients from errors and does not cause harm

	How **IMPORTANT** is this for supporting quality care in your setting? 1=Low, 5=High	How **FEASIBLE** is **measuring** this in your setting? (*e.g., % of patients, visits, care plans*) 1=Minimally, 5=Highly
**Clinical Management**		
Documenting a current medication list	1 2 3 4 5	1 2 3 4 5
Recording vital signs	1 2 3 4 5	1 2 3 4 5
Performing an exam for a presenting problem	1 2 3 4 5	1 2 3 4 5
Screening for signs and symptoms of serious pathology (i.e. red flags)	1 2 3 4 5	1 2 3 4 5
Screening for the possibility of pregnancy prior to obtaining radiographs	1 2 3 4 5	1 2 3 4 5
Referring patients at risk for self-directed violence to an appropriate provider	1 2 3 4 5	1 2 3 4 5
**Older Adults**		
Screening patients over age 40 for major risk factors for osteoporosis	1 2 3 4 5	1 2 3 4 5
Referring patients with new/recent osteoporotic fracture to a primary care or other relevant provider	1 2 3 4 5	1 2 3 4 5
Screening older adults for abilities to independently carry out activities of daily living	1 2 3 4 5	1 2 3 4 5
Offering older adults advice on balance, strength, and endurance exercises to prevent falls	1 2 3 4 5	1 2 3 4 5
**Outcomes related to patient safety**		
Documenting adverse events	1 2 3 4 5	1 2 3 4 5

This section contains 5 quality measures supporting care that are responsive to patient needs and preferences.

	How **IMPORTANT** is this for supporting quality care in your setting? 1=Low, 5=High	How **FEASIBLE** is **measuring** this in your setting? (*e.g., % of patients, visits, care plans*) 1=Minimally, 5=Highly
**Respect for patients**		
Completing an informed consent process before delivering care	1 2 3 4 5	1 2 3 4 5
Documenting patient involvement in care planning and decision-making	1 2 3 4 5	1 2 3 4 5
Number of days between request and an appointment	1 2 3 4 5	1 2 3 4 5
**Patient experience**		
Patients report involvement in care planning and decision-making	1 2 3 4 5	1 2 3 4 5
Patients report satisfaction with care	1 2 3 4 5	1 2 3 4 5

This section contains 10 quality measures supporting administrative characteristics of a health organization that support quality improvement. Please respond as they pertain to your organization.

	How **IMPORTANT** is this for supporting quality care in your setting? 1=Low, 5=High	How **FEASIBLE** is developing or documenting this in your setting? 1=Minimally, 5=Highly
**Administrative measures**		
Conducting regular audits to ensure regulatory compliance	1 2 3 4 5	1 2 3 4 5
Conducting regular audits are part of a quality control program	1 2 3 4 5	1 2 3 4 5
General employment training procedures	1 2 3 4 5	1 2 3 4 5
A reporting/supervisory structure	1 2 3 4 5	1 2 3 4 5
A future planning strategy	1 2 3 4 5	1 2 3 4 5
A current database of professional credentials for all providers	1 2 3 4 5	1 2 3 4 5
Infection control and prevention protocols	1 2 3 4 5	1 2 3 4 5
Training procedures for hand hygiene, protective equipment, and environmental cleaning	1 2 3 4 5	1 2 3 4 5
Securing patient records according to regulatory requirements	1 2 3 4 5	1 2 3 4 5
Costs of chiropractic care are transparent	1 2 3 4 5	1 2 3 4 5
