# Supplementary material for: Assessing the importance and feasibility of quality measures for chiropractic care: a national survey of U.S. chiropractors
Source: Chiropr Man Therap. 2026 Mar 28;34:18. doi: 10.1186/s12998-026-00635-1 (PMC13151159; doi:10.1186/s12998-026-00635-1)
Supplement: Supplementary file 2 — Supplementary Material 2: QM descriptions, metrics, rationale, and companion measures [file 12998_2026_635_MOESM2_ESM.pdf]

Supplemental table 1. Mean ratings for all rated measures by subgroup for importance and feasibility

Subgroup	Importance	Feasibility
Overall	4.26	4.07
Single Discipline	4.21	4.03
Multidisciplinary	4.49	4.30
Referral Only	4.45	4.31
Self-Referral	4.25	4.06
Metro/Micro	4.28	4.09
Small town/Rural	4.11	4.03
HPSA*	4.17	3.97
Not HPSA	4.27	4.09

*Health Professional Shortage Area

Supplemental table 2. Subgroup comparison of importance and feasibility rankings using Kendall's W (i.e. Kendall's coefficient of concordance)

Subgroups compared	Kendall's W* (95% CI)
Importance	
Single discipline vs. Multidisciplinary vs. Referral only	0.96 (0.73 to 1.00)
Feasibility	
Single discipline vs. Multidisciplinary vs. Referral only	0.89 (0.66 to 1.00)

*no agreement (.00-.09), very weak agreement (.10-.29), weak agreement (.30-.49), moderate agreement (.50-.69), strong agreement (.70-.89), and unusually strong agreement (.90-1.00)
