# Supplementary material for: Assessing the importance and feasibility of quality measures for chiropractic care: a national survey of U.S. chiropractors
Source: Chiropr Man Therap. 2026 Mar 28;34:18. doi: 10.1186/s12998-026-00635-1 (PMC13151159; doi:10.1186/s12998-026-00635-1)
Supplement: Supplementary file 3 — Supplementary Material 3: Supplemental tables [file 12998_2026_635_MOESM3_ESM.docx]

**Additional File 2**

**Quality measure (QM) descriptions, example metrics, rationale, and companion measures**

For organizations seeking to implement quality measures (QMs), each current QM is described with a brief title, brief description, example metric, and rationale. Successful QM implementation requires an understanding of how QMs of clinical processes, outcomes, and health organization characteristics (structural QMs) interrelate and support each other. To facilitate this understanding, the tables in this document identify companion measures which support, or are supported by, each measure.

QMs in this document are listed according to the domain to which they are most closely associated. The 3 domains are:

1. QMs supporting care that gets results (i.e., effective; efficient)

2. QMs supporting care that protects patients from error and does not cause harm (i.e., safe)

3. QMs supporting care that is responsive to patient needs and preferences (i.e., patient-centered, timely, and equitable).

There are currently 64 QMs, 31 of which describe clinical processes and outcomes. QMs of clinical processes and outcomes are ordered chronologically, generally following activities as they tend to occur during a health care visit. The final table includes 33 structural QMs, which describe health organization characteristics and infrastructure that support clinical processes and outcome measures.

**Table of** **Contents**

[Table 1. Clinical process and outcome measures supporting care that gets results 3](#_Toc206684141)

[Clinical evaluation 3](#_Toc206684142)

[Clinical screening 4](#_Toc206684143)

[Care planning 4](#_Toc206684144)

[Clinical outcomes 5](#_Toc206684145)

[Table 2. Clinical process and outcome measures supporting care that protects patients from errors and harm 6](#_Toc206684146)

[Clinical management 6](#_Toc206684147)

[Older adults 7](#_Toc206684148)

[Outcomes related to patient safety 7](#_Toc206684149)

[Table 3. Clinical process and outcome measures supporting care that is responsive to patients’ needs and preferences 8](#_Toc206684150)

[Respect for patients 8](#_Toc206684151)

[Patient experience 8](#_Toc206684152)

[Table 4. Structure measures supporting care that gets results 9](#_Toc206684153)

[Supports best practices 9](#_Toc206684154)

[Organizational framework 10](#_Toc206684155)

[Supports quality assessment/ improvement 10](#_Toc206684156)

[Table 5. Structure measures supporting care that protects patients from errors and harm 11](#_Toc206684157)

[Safety-related organizational infrastructure 11](#_Toc206684158)

[Table 6. Structure measures supporting care that is responsive to patients’ needs and preferences 13](#_Toc206684159)

[Supports timely access to care 13](#_Toc206684160)

[Supports autonomy and beneficence 13](#_Toc206684161)

[Protects privacy 14](#_Toc206684162)

[Supports a multidimensional-health approach 15](#_Toc206684163)

**Instructions:** Hover over the table title and click the arrow to expand or collapse. Click any heading or subheading in the Table of contents to navigate to that section.

# **Table 1. Clinical process and outcome measures supporting care that gets results**

| **Clinical evaluation** | | | | |
| --- | --- | --- | --- | --- |
| **Brief title** | **Brief description** | **Example metric** | **Rationale** | - **Corresponding QM** |
| Health history | Percentage of patients with documented past health history prior to initiating care | Numerator: # of visits for a new problem documenting past health history Denominator: # of visits for a new problem | Health history refers to past hospitalizations and other health-related events. Prior to initiating care, a past health history should be documented in the clinical record. Documenting past health history helps ensure pertinent information is available to inform a justified clinical evaluation and working diagnosis. | - Health history policy - Red flag screening policy |
| Condition specific history | Percentage of patients with documented condition-specific history prior to initiating care | Numerator: # of visits for a new problem documenting a condition-specific history; Denominator: # of visits for a new problem | Documenting a condition-specific history draws from the ethical principle of beneficence. The process offers pertinent information such as symptom onset, location, duration, progression, recurrence, variation, severity, and activity limitations. Obtaining a condition-specific history prior to initiating care helps ensure key information is available to inform and justify an appropriate clinical evaluation, working diagnosis, and care plan. | - Condition-specific history policy - Red flag screening policy |
| Review of systems | Percentage of patients whose clinical record includes a review of systems (e.g., cardiovascular, pulmonary, etc.) | Numerator: # of initial visits with a documented review of systems  Denominator: # of initial visits | Documenting a review of symptoms/problems associated with major body systems helps fill potential gaps in condition-specific and general health histories. Documenting a review of systems helps ensure pertinent information is available to inform a reasoned and justified clinical evaluation, working diagnosis, care plan, and to ensure safety. | - Health history policy |
| Outcome assessment (baseline) | Percentage of patients assessed with valid functional and/or symptom outcome measures as a baseline | Numerator: # of visits for a new problem that include a valid baseline outcome assessment  Denominator: # of visits for a new problem | Clinical guidelines, care standards, and best practices recommendations for chiropractic care consistently suggest measuring clinical outcomes with valid instruments, beginning with obtaining baseline measures when care is initiated for a new problem. Currently, there is no consensus on which validated outcome instruments are the most important. | - Patient experience data collection - Re-evaluation policy |
| Response to care | Percentage of patients whose response to care is regularly assessed | Numerator: # of follow-up visits documenting response to prior care  Denominator: # of follow-up visits in a care plan | Assessing response to care is based on a care standard that draws from the ethical principle of beneficence. Regularly assessing response to care is a process that gathers information to inform and ensure timely clinical management decisions such as discharge, continued care, and referral. | - Re-evaluation policy |
| Additional care assessment | Percentage of visits where the need for additional visits is assessed | Numerator: # of visits documenting an assessment for additional visits Denominator: # of visits | Providing unneeded care places patients at greater risk for dependency on providers, adverse events and financial burden. Providing unneeded care also reduces the availability of health care services for those in need, and the effectiveness of care. | - Re-evaluation policy |
| **Clinical screening** | | | | |
| Psychosocial risk factor screening | Percentage of patients assessed for psychological and social risk factors for poor outcome and/or chronicity | Numerator: # of visits for a new problem with psychosocial screening Denominator: # of visits with a new problem | Symptoms are influenced by many potential factors. Therefore, it is important to consider factors which directly and indirectly contribute to symptom severity, chronicity, and reduce self-efficacy or capacity to self-monitor and self-manage. Screening is a process designed to help identify relevant psychological and social factors that can negatively influence a person’s capacity to improve so they can be subsequently addressed. | - Psychosocial risk factor screening policy - Psychosocial risk factor intervention infrastructure - Mind-body resources |
| Opioid use screening | Percentage of patients screened for opioid use | Numerator: # of patients screened for opioid use Denominator: # of patients | Screening for opioid use relates to the role chiropractic practitioners have in providing health promotion and clinical preventive services. Screening is recommended because of the high prevalence of opioid use and potential negative health ramifications such as greater risks for dependence and suicide. Screening helps inform the potential need to collaborate with prescribers toward the goal of reducing opioid use and preventing or reducing dependence. | - Health history policy |
| Tobacco use screening | Percentage of patients screened for tobacco use | Numerator: # of patients screened for tobacco use Denominator: # of patients | Tobacco use increases risk factors for numerous chronic diseases. Screening adolescents and adults for tobacco use offers key information to inform the health history, clinical evaluation, and management decisions. Screening identifies patients for whom it is appropriate to offer information about negative health effects related to tobacco use and a variety of cessation interventions. Successful tobacco cessation lowers the risk for heart disease, lung disease, and stroke. | - Health history policy |
| Physical activity screening | Percentage of patients screened for physical activity level | Numerator: # of patient visits for a new problem with physical activity screening Denominator: # of visits with a new problem | Physical activity screening is a best practice recommendation. Practitioners have a role in health promotion and clinical prevention by offering assessment and informed advice. Physical activity carries few risks and multiple health benefits including reduced risk for common chronic diseases. | - Health history policy - Self-management policy - Activity advice policy |
| **Care planning** | | | | |
| Current care plans | Percentage of visits with a current care plan | Numerator: # of visits with a current care plan Denominator: # of visits | The purpose of this measure is to ensure a plan of care on any given visit is present in the clinical record. Current care plans can help ensure both patients and providers are aware of the plan. Current plans also help ensure continuity of care when performed by more than 1 provider, and they can meaningfully inform other providers offering concurrent care. | - Continuity of care policy - Re-evaluation policy |
| Care plans | Percentage of care plans based on a clinical evaluation | Numerator: # of visits with a current care plan Denominator: # of visits | Care plans document a planned course of care. Clinical evaluations offer up-to-date information about a working diagnosis, patient goals and preferences. The purpose of the measure is to ensure all care plans (initial or subsequent) are based on a clinical evaluation to provide the information needed to appropriately justify care. | - Examination policy |
| Multimodal care plans | Percentage of care plans including 5 components: 1) Active therapies such supervised or unsupervised exercise; 2) Manual therapies such as joint manipulation and myofascial therapies; 3) Education about one's condition including pain physiology when appropriate; 4) Self-management advice and/or activities; and 5) Therapeutic goals. *Multimodal interventions are not required at each visit during a care plan | Numerator: # of care plans including Active therapies; Manual therapies; Education; Self-management advice and/or activities; Goals Denominator: # of care plans | Symptoms are influenced by many potential factors. Therefore, it is important to consider factors which directly and indirectly contribute to symptom severity, chronicity, and reduce self-efficacy or capacity to self-monitor and self-manage. Active therapies are those in which patients take an active role in performing, such as exercise. Manual therapies are primarily performed by providers. Education is a process of helping patients understand a condition, how treatment addresses the problem, how and when to seek care, and how to better self-monitor and interpret symptoms. Self-management advice and/or activities are focused on building and reinforcing self-efficacy and self-management capacity and preventing unneeded dependence on providers. Therapeutic goals facilitate patient engagement in care planning and aid in clinical re-evaluation. Because 1 or more components may not be appropriate for every care plan, documenting why a component is excluded clarifies that each component was thoughtfully considered. The example metric currently measures a dichotomous outcome (e.g., all categories are included, or all categories are not included). A metric assessing the number of components included may be more appropriate. | - Multimodal care plan policy - Self-management policy - Activity advice policy |
| **Clinical outcomes** | | | | |
| Outcome assessment (re-evaluation) | Percentage of patients assessed during a re-evaluation using a valid functional and/or symptom outcome measure | Numerator: # of re-evaluation visits including a valid outcome assessment instrument  Denominator: # of re-evaluation visits | Assessing response to care with validated tools offers objective evidence about the effectiveness of care. Currently, there is no gold-standard functional or symptom outcome instrument. Instead, many possible instruments can be used, depending on body region, functional limitation, or other associated symptoms or problems (e.g., pain severity, pain interference). | - Re-evaluation policy - Patient experience policy |
| Return to work time | Return to work time for patients with a work-related injury | Mean or median # of days before non-working patients, treated for a work-related injury, returned to work | Returning to work is a meaningful and measurable timepoint that relates to the effectiveness of care. A lower calculated performance time (measured in days, weeks, or months) for this measure indicates higher quality care. | - Patient experience data collection |

# **Table 2. Clinical process and outcome measures supporting care that protects patients from errors and harm**

| **Clinical management** | | | | |
| --- | --- | --- | --- | --- |
| **Brief title** | **Brief description** | **Example metric** | **Rationale** | **Corresponding QM** |
| Current medication list | Percentage of patients with a documented list of current medications | Numerator: # of patient records with a current medication list  Denominator: # of patient records | Documenting current medications helps ensure vital information is available to inform a clinical evaluation, potential need for referral, working diagnosis, and decisions about safety. | - Health history policy - Safety and risk management policy |
| Vital signs | Percentage of patients whose vital signs are recorded | Numerator: # of visits for a new problem with documented vital signs Denominator: # of visits for a new problem | Vital signs such as temperature, heart rate, respiratory rate, weight, height, and blood pressure can be used to help identify potentially serious pathology and support management decisions. It is yet to be determined how often vital signs should be recorded to represent a component of quality. | - Red flag screening policy - Safety and risk management policy - Referral system |
| Examination | Percentage of patients who receive a clinical exam for a presenting problem | Numerator: # of visits for a new problem with a corresponding examination for the problem; Denominator: # of visits for a new problem | Clinical guidelines and best practice recommendations consistently state physical examination, appropriate to the complexity of a presenting problem, is a critical component of chiropractic clinical management and an ethical responsibility. Examination is needed to identify potentially serious pathology and to inform working diagnoses and subsequent clinical management decisions. | - Examination policy - Red flag screening policy - Safety and risk management policy - Referral system |
| Red flag screening | Percentage of patients screened for signs and symptoms of serious pathology (i.e. red flags) | Numerator: # of visits for a new problem with screening for serious pathology; Denominator: # of visits for a new problem | Clinical guidelines, best practice recommendations and care standards consistently recommend screening for signs and symptoms of conditions indicating potential serious pathology. Screening information helps ensure safety by informing decisions about diagnostic testing, treatment, monitoring, and referral. | - Red flag screening policy - Safety and risk management policy - Referral system - Interprofessional collaboration policy |
| Radiographic screening | Percentage of patients screened for the possibility of pregnancy prior to obtaining radiographs | Numerator: # of radiographic exams where the possibility of pregnancy is documented Denominator: total # of radiographic exams | Screening patients for the possibility of pregnancy prior to obtaining radiographs is a process focused on preventing avoidable harm to a fetus. Information obtained from screening can inform a clinical benefit vs. fetal risk analysis, which can then be used to either justify or avoid imaging, consistent with radiographic guidelines. | - Safety and risk management policy - Diagnostic imaging policy |
| Referral to prevent self-directed violence | Percentage of patients at risk for self-directed violence referred to an appropriate provider | Numerator: # of patients identified as at risk for self-directed violence, referred to an appropriate provider  Denominator: # of patients identified as at risk for self-directed violence | A fundamental responsibility for chiropractic providers is to help ensure the safety of patients. This measure is derived from a best practice recommendation focused on ensuring people at risk for self-directed violence are promptly referred to professionals specifically trained and experienced in addressing related thoughts and behaviors. | - Self-directed violence prevention referral pathway - Referral system - Interprofessional collaboration policy - Psychosocial risk factor intervention infrastructure |
| **Older adults** | | | | |
| Osteoporosis risk factor screening | Percentage of patients over age 40 screened for major risk factors for osteoporosis | Numerator: # of patients over age 40 assessed for major risk factors for osteoporosis  Denominator: # of patients over age 40 | Though osteoporosis is more commonly observed in postmenopausal women over the age of 50, screening earlier theoretically offers additional opportunity to prevent osteoporosis. Screening for major risk factors for osteoporosis can be performed by obtaining a detailed health and family history and using online risk calculators such as QFracture (https://qfracture.org/) and FRAX (https://frax.shef.ac.uk/FRAX/). Bone mineral density tests are second-tier studies designed to confirm and quantify bone density for people previously identified at higher risk. | - Safety and risk management policy - Referral system - Interprofessional collaboration policy |
| Osteoporotic fracture referral | Percentage of patients with new/recent osteoporotic fracture referred to a primary care or other relevant provider | Numerator: # of patients with new/recent osteoporotic fracture referred to a primary care or other relevant provider  Denominator: # of patients with new/recent osteoporotic fracture | Specialty care may be needed to comprehensively assess and manage osteoporotic fractures and future risk. | - Referral system - Interprofessional collaboration policy |
| Older adult functional health screening | Percentage of older adults screened for abilities to independently carry out activities of daily living | Numerator: # of older adults screened for functional health status  Denominator: # of older adults | Aging adults are more likely to experience challenges with hearing, sight, coordination, and cognition that can negatively affect the capacity to carry out activities of daily living. Activities of daily living include bathing, toileting, eating, and walking. Higher-level functional activities include tasks like cooking, writing, and driving. People with difficulty carrying out 1 or more activities of daily living may be at risk for other health-related problems. Identifying individuals who have difficulty performing these activities gives providers an opportunity to offer appropriate advice and/or refer to an appropriate specialist or community resource. | - Older adult functional health screening policy - Referral system - Interprofessional collaboration policy |
| Fall prevention (older adults) | Percentage of older adults offered advice on balance, strength, and endurance exercises to prevent falls | Numerator: # of older adult patients offered advice on exercises to prevent falls Denominator: # of older adult patients | Aging adults are more likely to experience challenges with balance, strength, flexibility, and endurance, which can increase risk for falls and subsequent morbidity. Offering advice on these activities is considered a best practice focused on preventing negative fall-related health consequences. | - Self-management policy - Activity advice policy - Multimodal care plan policy |
| **Outcomes related to patient safety** | | | | |
| Adverse event rate | Percentage of adverse events while receiving or immediately after receiving chiropractic care (e.g., increased pain, stiffness, dizziness, headache) | Numerator: # of adverse events  Denominator: # of patient visits | Health care providers have a responsibility to help ensure that appropriate procedures are in place to reduce/eliminate avoidable harm to patients and to offer data to better inform consent processes. | - Safety and risk management policy - Patient experience data collection |

# **Table 3. Clinical process and outcome measures supporting care that is responsive to patients’ needs and preferences**

| **Respect for patients** | | | | |
| --- | --- | --- | --- | --- |
| **Brief title** | **Brief description** | **Example metric** | **Rationale** | **Corresponding QM** |
| Appointment wait time | Number of days between request and an appointment for chiropractic care | Median # of days between appointment request and appointment | This measure demonstrates how effective an organization is in offering timely access to care. Timeliness is one of 6 quality domains described by the Agency for Healthcare Research and Quality. Timely access to care prevents harmful delays that can contribute to chronicity and worsening symptoms. Timely access also offers an early opportunity to identify and address serious pathology. | - Appointment wait time policy |
| Informed consent process | Percentage of patients receiving care only after completing an informed consent process | Numerator: # of care plans documenting informed consent from patient, parent, or guardian  Denominator: # of care plans | Providers have an ethical responsibility to ensure people are informed about their care and offer consent before any care is administered. The concept of informed consent extends beyond a single timepoint requiring an ongoing process. This measure is aligned with the patient-centered domain of quality to help ensure care that is respectful of and responsive to individual patient preferences, needs, and values. | - Informed consent policy - Explanation policy - Shared decision making policy |
| Shared decision-making process | Percentage of patients involved in care planning and decision-making | Numerator: # of patients involved in care planning and decision-making Denominator: # of patients with care plans | This measure is focused on providing care that is respectful of and responsive to individual patient preferences, needs, and values and ensuring patient values guide all clinical decisions. The metric for this measure represents a clinical process led by providers and documented in the healthcare record. A separate measure assesses the effectiveness of this process (percentage of patients reporting involvement in their care planning and decision-making). | - Shared decision-making policy - Self-management policy |
| **Patient experience** | | | | |
| Shared decision-making outcome | Percentage of patients reporting involvement in their care planning and decision-making | Numerator: # of patient surveys reporting shared decision-making was part of the care planning process Denominator: # of patients responding to questions about involvement in care planning and decision-making | This measure aligns with the patient-centered quality domain, which is focused on providing care that is respectful of and responsive to individual patient preferences, needs, and values. A separate measure assesses the extent to which providers document patient involvement in care planning and decision-making. This measure is derived from patient surveys reflecting the effectiveness of shared decision-making processes. | - Shared decision-making policy - Self-management policy - Patient experience data collection |
| Patient satisfaction | Percentage of patients reporting satisfaction with care | Numerator: # of patients reporting satisfaction with care  Denominator: # of patients responding to questions about satisfaction | Satisfaction is a general term which may relate to any quality domain (Safe, Effective, Patient-centered, Timely, Efficient, Equitable). The current measure generally assesses if care meets people’s needs. However, quality measures used by other professions measure key aspects of patient experience rather than general satisfaction. Until patient experience measures can replace this measure, health organizations may wish to assess more detailed satisfaction with, for example, scheduling processes, interpersonal interactions, access, timeliness of care, and cleanliness. | - Patient experience data collection |

# **Table 4. Structure measures supporting care that gets results**

| **Supports best practices** | | | | |
| --- | --- | --- | --- | --- |
| **Brief title** | **Brief description** | **Example metric** | **Rationale** | **Corresponding QM** |
| Health history policy | The organization requires obtaining a health history prior to clinical evaluation and management | Policy requiring a documented health history for a problem being evaluated/managed | Organizational support is needed to facilitate the clinical process of obtaining and recording a health history for the purpose of ensuring pertinent information is available to inform a justified clinical evaluation, working diagnosis, and patient safety. | - Health history - Review of systems - Opioid use screening - Physical activity screening - Tobacco use screening - Current medication list |
| Condition-specific history policy | The organization requires obtaining a condition specific history prior to clinical evaluation and management | Policy requiring a documented condition specific history for a problem being evaluated/managed | Organizational support for the clinical process of obtaining and recording a condition-specific history is needed to ensure pertinent information is available to inform a justified and safe clinical evaluation and working diagnosis and care plan. | - Condition specific history |
| Psychosocial risk factor screening policy | The organization supports screening for psychosocial risk factors for poor outcome and/or chronicity | Policy designating psychosocial risk factor screening | This measure documents organizational support for the clinical process of screening to identify relevant psychological and social factors negatively influencing a person. | - Psychosocial risk factor screening |
| Diagnostic imaging policy | The organization supports diagnostic imaging consistent with clinical guidelines and best practices | Policy and practical resources to guide diagnostic imaging use | An organization’s support for guideline-congruent diagnostic imaging practices helps ensure safety, costs, and other harms by reducing unnecessary and wasteful imaging. | - Radiographic screening |
| Re-evaluation policy | The organization expects regular monitoring and re-evaluation to inform discharge, continued care, and referral decisions | Policy expecting regular re-evaluation of ongoing care to inform decisions for discharge, continued care, or referral | Regularly assessing care response helps inform timely management decisions such as the need for additional visits and preventing unneeded care through discharge, and referral. | - Response to care - Outcome assessment (re-evaluation) - Current care plans - Additional care assessment |
| Multimodal care plan policy | The organization supports multimodal chiropractic care | Policy requiring care plans with designated components | Organizational support for multimodal chiropractic care is demonstrated by policy and procedures that encourage and support providers in employing interventions that address symptoms, behaviors, beliefs, knowledge, skills, and other factors that influence symptoms and a person’s capacity to self-monitor and self-manage a problem. | - Multimodal care plans - Fall prevention (older adults) |
| Activity advice policy | The organization supports advice to stay active for patients with low back pain | Policy supporting advice to stay active for patients with low back pain | This measure sets expectations for providers to offer advice to stay active. | - Multimodal care plans |
| **Organizational framework** | | | | |
| Employee training | The organization has employment training procedures | Documentation of employment training procedures | The presence of structured new employee training procedures represents a practical application of a strategy that seeks to maximize the provision of high-quality care. | - This is a general measure demonstrating organizational commitment to supporting quality care |
| Organizational structure | The organization has a reporting / supervisory structure | Documentation of organizational structure | A coordinated organizational structure that includes supervision and clear lines of responsibility offers a framework that can help employees work efficiently toward maximizing the provision of high-quality care. | - This is a general measure demonstrating organizational commitment to supporting quality care |
| Organizational planning | The organization has a future planning strategy | Documentation of a future planning strategy | Organizations seeking to avoid the status quo are focused on improvement through future planning because patient, employee, and community needs can change over time. | - This is a general measure demonstrating organizational commitment to supporting quality care |
| Provider credential database | The organization maintains a current database of professional credentials for all providers | Database documenting current professional credentials of all providers | A provider credential database offers information on available expertise (and gaps) that can influence quality of care. A database, for example, reviewed in concert with internal referral pathways may identify the need to hire providers with specific training and/or expertise. Alternatively, a database can inform an organization of the need to develop an external referral pathway(s). | - This is a general measure demonstrating organizational commitment to supporting quality care |
| **Supports quality assessment/ improvement** | | | | |
| Regulatory compliance audits | The organization conducts regular audits to ensure regulatory compliance | Policy designating periodic compliance audits | Regular auditing is a standard practice across health organizations. Regulatory compliance helps ensure patient and employee safety and acts to identify and address factors to prevent unintended harm. | - This is a general measure demonstrating organizational commitment to supporting quality care |
| Quality control audits | The organization conducts regular audits as part of a quality improvement program | Results of completed quality control audits | Health organizations conducting regular audits as part of a quality improvement program demonstrate the practical application of, and a sustained commitment to, quality improvement. | - This is a general measure demonstrating organizational commitment to supporting quality care |

# **Table 5. Structure measures supporting care that protects patients from errors and harm**

| **Safety-related organizational infrastructure** | | | | |
| --- | --- | --- | --- | --- |
| **Brief title** | **Brief description** | **Example metric** | **Rationale** | **Corresponding QM** |
| Infection control protocols | The organization has infection control and prevention protocols | Documentation of infection control protocols | Infection control and prevention protocols demonstrate a commitment to prevent unintended harm. | - This is a general measure demonstrating organizational commitment to supporting quality |
| Infection control training | Training procedures are in place for hand hygiene, personal protective equipment, and environmental cleaning | Documentation of hygienic training procedures | Training procedures focused on hand hygiene, personal protective equipment and environmental cleaning are needed to ensure employees engage in activities that prevent harm to both patients and employees | - This is a general measure demonstrating organizational commitment to supporting quality care |
| Red flag screening policy | Screening for serious pathology (i.e., red flags) is expected by the organization | Policy requiring screening for serious underlying pathology | Screening for serious pathology is a necessary part of a responsible diagnostic process. Information gained can inform subsequent decisions about diagnostic testing, treatment, monitoring, and referral. | - Red flag screening - Vital signs - Health history - Condition specific history - Examination |
| Self-directed violence prevention referral pathway | A referral pathway is in place for patients at risk for self-directed violence | Documentation of a referral pathway for patients with increased risk for self-directed violence | A referral pathway for patients at risk for self-directed violence is needed to ensure at risk individuals are connected with professionals specifically trained and experienced in addressing related thoughts and behaviors. | - Referral to prevent self-directed violence |
| Safety and risk management policy | The organization prioritizes safety through incident reporting, clinical risk management, and adverse event reporting | Policy and procedures to support a culture of safety including incident reporting, clinical risk management, and adverse event reporting | Organizations seeking to support a culture of safety establish guidance (i.e., policy) that supports adverse event, safety incident, and safety risk reporting. | - Red flag screening - Radiographic screening - Osteoporosis risk factor screening - Examination - Current medication list - Vital signs - Adverse event rate - Infection control training - Infection control protocols - Employee training |
| Referral system | A referral pathway is in place for patients with signs and/or symptoms of conditions outside the scope of chiropractic care | Documented referral pathways for patients needing services from specialists | Established referral pathways enable providers with a process designed to help connect patients with other providers with the appropriate training and expertise for a health need. | - Osteoporosis risk factor screening - Osteoporotic fracture referral - Red flag screening - Examination - Referral to prevent self-directed violence - Older adult functional health screening |
| Examination policy | The organization expects examination of a body region prior to delivering care to the area | Policy directing clinical evaluation prior to engaging in clinical interventions | Physical examination, appropriate to the complexity of a presenting problem, is a critical component of chiropractic clinical management and an ethical responsibility of providers. This measure communicates expectations of health organizations through guidance (i.e., policy). | - Examination - Care plans |
| Older adult functional health screening policy | The organization supports screening older adults for abilities to carry out activities of daily living | Policy and procedure(s) supporting the screening of older adults for the capacity to carry out daily living activities | Aging adults are more likely to experience challenges with hearing, sight, coordination, and cognition that negatively affect the capacity to carry out activities of daily living. This measure expresses institutional guidance and support that communicates expectations of providers and provides procedures to conduct screening. | - Older adult functional health screening |

# **Table 6. Structure measures supporting care that is responsive to patients’ needs and preferences**

| **Supports timely access to care** | | | | |
| --- | --- | --- | --- | --- |
| **Brief title** | **Brief description** | **Example metric** | **Rationale** | **Corresponding QM** |
| Appointment wait time policy | The organization supports scheduling timely chiropractic care | Organizational guidance and infrastructure supporting the timely scheduling of visits | Timeliness is a key quality domain. Timely access to care prevents harmful delays that can contribute to chronicity and worsening symptoms, offering early opportunity to identify and address serious pathology. The measure may exist as policy-level documents offering guidance about performance expectations, efficient methods to support scheduling, and regular needs assessments related to: patient population, demand for services, available providers, equipment, physical space, etc. | - Appointment wait time |
| **Supports autonomy and beneficence** | | | | |
| Informed consent policy | The organization requires informed consent from a patient, parent, or legal guardian prior to delivering clinical services | Policy requiring informed consent from patients, parents, or legal guardians, prior to delivering clinical services | The measure confirms institutional expectations and support for care delivered only after informed consent is obtained. The measure can be demonstrated through policy-level documents that communicate expectations for consent, and procedures for engaging in and efficiently documenting a consent process. | - Informed consent process |
| Explanation policy | The organization expects providers to communicate an explanation of the suspected/confirmed condition to patients | Policy requiring providers to communicate an explanation of their suspected/confirmed condition | Making informed decisions about care requires an understanding of one’s condition, how treatments interact with a condition, potential benefits and risks, and options. This measure helps ensure a valid informed consent process occurs and that care is respectful of and responsive to individual patient preferences and needs. | - Informed consent process |
| Continuity of care | The organization supports continuity of ongoing care with the same provider during e course of a care plan | Policy and infrastructure supporting care delivered to patients by the same provider | Organizations supporting continuity prioritize care that avoids wasting equipment, time, and ideas. Higher continuity is related to lower mortality and higher patient satisfaction in some settings. The measure can be demonstrated through policy-level documents communicating expectations, scheduling methods, full staff employment levels, and scheduling provider shifts to promote continuity. | - Current care plans |
| Cost transparency | Costs of chiropractic care are transparent | Evidence of public access (e.g., website) to costs of initial and follow-up chiropractic visits, or costs for chiropractic services | Cost transparency demonstrates an organization’s support for offering information to patients about the financial burdens associated with receiving evaluation and care. Cost transparency enables patients to make informed cost-benefit decisions about receiving services. | - This is a general measure demonstrating organizational commitment to supporting quality care |
| Shared decision-making policy | The organization supports shared decision-making among providers and patients | Policy requiring providers to engage in shared decision-making in the care planning process | The measure demonstrates a health organization’s commitment to supporting care that is respectful of and responsive to individual patient preferences, needs, and values and ensuring patient values guide all clinical decisions. Guidance sets expectations for shared decision-making and compels organizations to provide the training and infrastructure needed to support related clinical processes. | - Informed consent process - Shared decision-making process - Shared decision-making outcome |
| Interprofessional collaboration policy | The organization recognizes the responsibility of providers to refer, co-manage, and/or communicate with other healthcare professionals when clinically indicated and authorized by patients | Policy supporting collaboration with other healthcare professionals when indicated | Referral is a process for facilitating the right care, at the right time, and from an appropriately trained and experienced provider. | - Red flag screening - Osteoporosis risk factor screening - Osteoporotic fracture referral - Older adult functional health screening - Referral to prevent self-directed violence - Psychosocial risk factor screening |
| Self-management policy | The organization encourages interventions to support patient self-management capacity such as education and active care approaches | Policy supporting improving self-management capacity among patients | The prolonged use of passive interventions can contribute to preventable dependency on providers and/or their services. | - Fall prevention (older adults) - Multimodal care plans - Shared decision-making process - Shared decision-making outcome |
| Patient experience data collection | The organization collects data from patients about their care | Data from patients about the experience of receiving care | Patients are the most important stakeholders in health care, and their data represents key information regarding clinical outcomes, the experience of care, and clinical and clerical processes used by health organizations. | - Outcome assessment (baseline) - Outcome assessment (re-evaluation) - Shared decision-making outcome - Patient satisfaction - Return to work time   Adverse event rate |
| **Protects privacy** | | | | |
| Records management policy | The organization secures patient records according to regulatory requirements | Policy designating compliance with regulatory requirements for health record privacy and security | A core responsibility of a health organization is to protect patients. This responsibility extends to protecting records that contain sensitive information regulated by regional and/or national level regulations. | - This is a general measure demonstrating organizational commitment to supporting quality care |
| **Supports a multidimensional-health approach** | | | | |
| Psychosocial risk factor intervention infrastructure | The organization maintains the capacity to connect patients with psychological resources (e.g., mental health providers) and social resources (e.g., community groups, religious organizations) | Evidence of referral pathways and other community resources to support psychological and social health | Screening for meaningful psychosocial risk factors is not useful if needs are not addressed. This measure represents the organizational infrastructure that facilitates connecting patients with needed resources when identified through formal screening or other methods. | - Psychosocial risk factor screening - Referral to prevent self-directed violence |
| Mind-body resources | Resources are available for mind-body interventions such as Cognitive-Behavioral Therapy and Mindfulness-Based Stress Reduction | Documentation of patient-accessible mind-body resources | Maintaining resources for connecting patients with these interventions is consistent with best practice recommendations and a person-centered approach recognizing pain is influenced by many factors that can extend beyond anatomical/biological explanations. | - Psychosocial risk factor screening |
